# Endemicity, Biogeography, Composition, and Community Structure On a Northeast Pacific Seamount

**DOI:** 10.1371/journal.pone.0004141

**Published:** 2009-01-07

**Authors:** Craig R. McClain, Lonny Lundsten, Micki Ream, James Barry, Andrew DeVogelaere

**Affiliations:** 1 Monterey Bay Aquarium Research Institute, Moss Landing, California, United States of America; 2 Department of Biology, Stanford University, Stanford, California, United States of America; 3 Monterey Bay National Marine Sanctuary, Monterey, California, United States of America; University of Bristol, United Kingdom

## Abstract

The deep ocean greater than 1 km covers the majority of the earth's surface. Interspersed on the abyssal plains and continental slope are an estimated 14000 seamounts, topographic features extending 1000 m off the seafloor. A variety of hypotheses are posited that suggest the ecological, evolutionary, and oceanographic processes on seamounts differ from those governing the surrounding deep sea. The most prominent and oldest of these hypotheses, the seamount endemicity hypothesis (SMEH), states that seamounts possess a set of isolating mechanisms that produce highly endemic faunas. Here, we constructed a faunal inventory for Davidson Seamount, the first bathymetric feature to be characterized as a ‘seamount’, residing 120 km off the central California coast in approximately 3600 m of water (Fig 1). We find little support for the SMEH among megafauna of a Northeast Pacific seamount; instead, finding an assemblage of species that also occurs on adjacent continental margins. A large percentage of these species are also cosmopolitan with ranges extending over much of the Pacific Ocean Basin. Despite the similarity in composition between the seamount and non-seamount communities, we provide preliminary evidence that seamount communities may be structured differently and potentially serve as source of larvae for suboptimal, non-seamount habitats.

## Introduction

If a species on Earth were selected at random, would its range be small and confined to a specific locality, or would that species be distributed broadly across continents and oceans? Despite the complex interplay of historical accidents, climatic and oceanographic forces, and the biological traits of the species themselves, similarities exist in the distribution of biogeographic ranges among taxa[1]–[7]. In birds, mammals, and insects, the frequency distribution of range sizes tends to be unimodal and right-skewed (i.e., most species have relatively restricted ranges)[6], [7]. In contrast, the ranges for marine organisms are thought to be broad due the apparent scarcity of physical or physiological isolating barriers in the open ocean [8], [9]. Yet for many marine groups a significant proportion of species (10–70%) possess narrow geographic ranges, challenging such hypotheses[9].

In the deep sea, the perceived homogeneity of seafloor habitats with relatively little environmental variation (e.g., temperature, salinity, and pressure) has lead to the conjecture that species have broad horizontal ranges[10]. However, because abiotic and biotic factors do vary greatly with depth, many species often possess restricted vertical ranges despite this potential for broad horizontal ranges[11]. Allen & Sanders[12] noted that approximately 50% of North Atlantic bivalves possessed geographic distributions that included the entire basin, a finding echoed by Rex et al.[13] for gastropods. In contrast, seamounts, underwater mountains with summits below the ocean surface, are thought to possess a set of isolating mechanisms that produce highly endemic faunas[14]–[16]. Oceanic currents that trap larvae on seamounts, the presence of unique or rare deep-sea habitats such as hard rock substrate and coral/sponge reefs, among other hypotheses, are thought to lead to genetic isolation[15], [17], an idea questioned by some[18]. This perceived endemicity is at least part of major initiatives to characterize and conserve these potential biodiversity hotspots (IUCN, EU, WCPA, WWF, Seamounts Online, CenSeam). However, new studies demonstrating that specific faunal components are composed of non-endemic species are challenging this idea[19], [20]. We refer to this hypothesis that seamounts are ecologically and evolutionary isolated from other deep-sea habitats and, therefore contain high levels of endemic species as the seamount endemicity hypothesis (abbreviated here as the SMEH).

Here, we construct a megafaunal inventory for Davidson Seamount off the central California coast based on six expeditions and over 60000 faunal observations. Utilizing additional data collected over the past 25 years by the Monterey Bay Aquarium Research Institute combined with a survey of the literature, we assess the current rates of endemicity and biogeographic ranges for the megafaunal assemblage occurring on this seamount. Overall we find little evidence to support SMEH and instead document an assemblage of cosmopolitan species similar to other deep-sea benthic habitats. We do however, find, evidence that biological communities on seamounts are structured differently when compared to other deep-sea habitats despite similarities in species composition.

## Results

An examination of the species accumulation curve for Davidson Seamount indicates the locality is relatively well sampled and that future sampling efforts are unlikely to uncover many new species (Fig. 2). Currently, we have identified 168 species of megafauna from this seamount. Overall, we find little evidence of endemicity (≤7%) at Davidson (i.e., species unique to Davidson Seamount specifically; Fig. 3). We are confident that 71% of the species are cosmopolitan (i.e., distributed on seamounts and other non-seamount habitats). In addition, sufficient data exists for 22% of the observed species to suggest strongly that their ranges are not limited to seamounts. The remaining 7%, 12 species, were identified solely from video observations of their morphology, since no specimens were collected. Explicit species assignments for these organisms, including 3 holothuroids, 1 demosponge, and 8 hexactinellid sponges were not possible, yet they may be known species with biogeographic ranges beyond Davidson Seamount. For example, because spicule samples used normally for poriferan taxonomy were not available, the 8 hexactinellids were assigned to individual morphotypes that may or may not represent known species. However, many of the remaining sponge species identified here do have very large geographic ranges, including those species new to science and recently described based on specimens collected at Davidson Seamount[21], suggesting that endemicity is not typical of the group.

**Figure 1:**
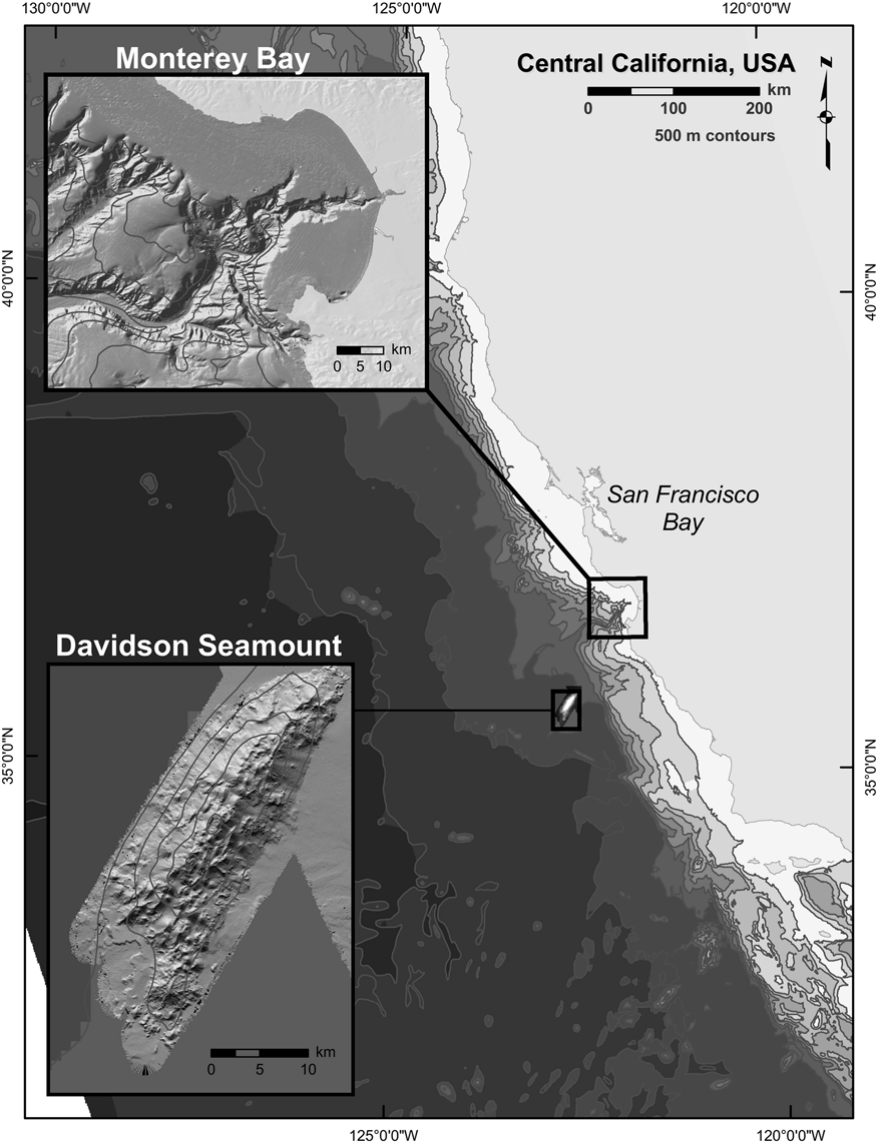
Bathymetric map of the Central California Coast with Monterey Canyon and Davidson Seamount.

**Figure 2:**
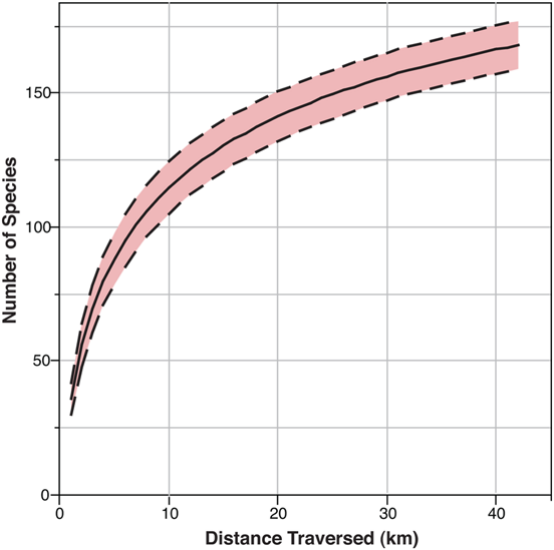
Species accumulation curve (Mao Observed) for distance traversed at Davidson Seamount. Dotted lines indicate 95% confidence intervals.

**Figure 3:**
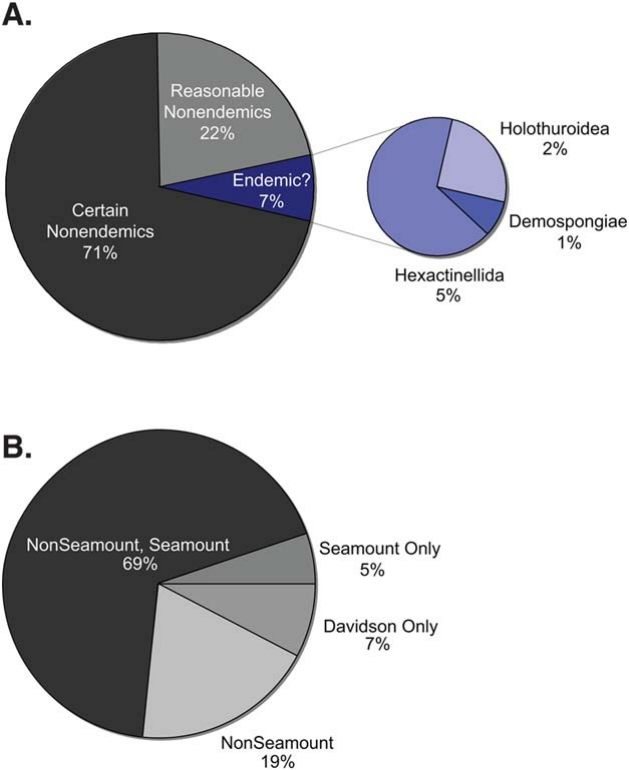
Pie charts A. illustrating the percentage of species potentially endemic to Davidson and taxonomic makeup of those species and B. the percentage of species at Davidson found in different seamount and nonseamount habitats.

McClain[18] advocated that discussions of seamount biodiversity define the spatial scale and grain of endemicity. Specifically, 1) the number of species found only on seamounts 2) number of species found only on a particular seamount chain, 3) the number of species found only on individual seamounts, 4) the number of species restricted to a particular habitat on a seamount, and 5) the number of species found in a single sample, among replicate samples, on a single seamount chain. Twelve percent of the species found at Davidson are confined to local seamounts (scale 1). However, this 12% estimate includes the 12 species discussed above (endemic to Davidson Seamount, scale 3) and the true percentage endemic to seamounts alone may actually be lower. Of all the species found at Davidson, 88% (146 species) have also been observed in non-seamount habitats along the continental margin. Interestingly, 19% (31 species) of the species at Davidson Seamount, although found on continental margins (locally or globally) are unknown from other seamounts. Insufficient data is available to determine the extent that species are restricted to particular habitats on Davidson Seamount or solely to Northeast Pacific seamounts. With regard to the number of species found in single samples, 9% of the species found have been observed fewer than 10 times on Davidson Seamount with 3.5% limited to single video observations.

**Figure 4:**
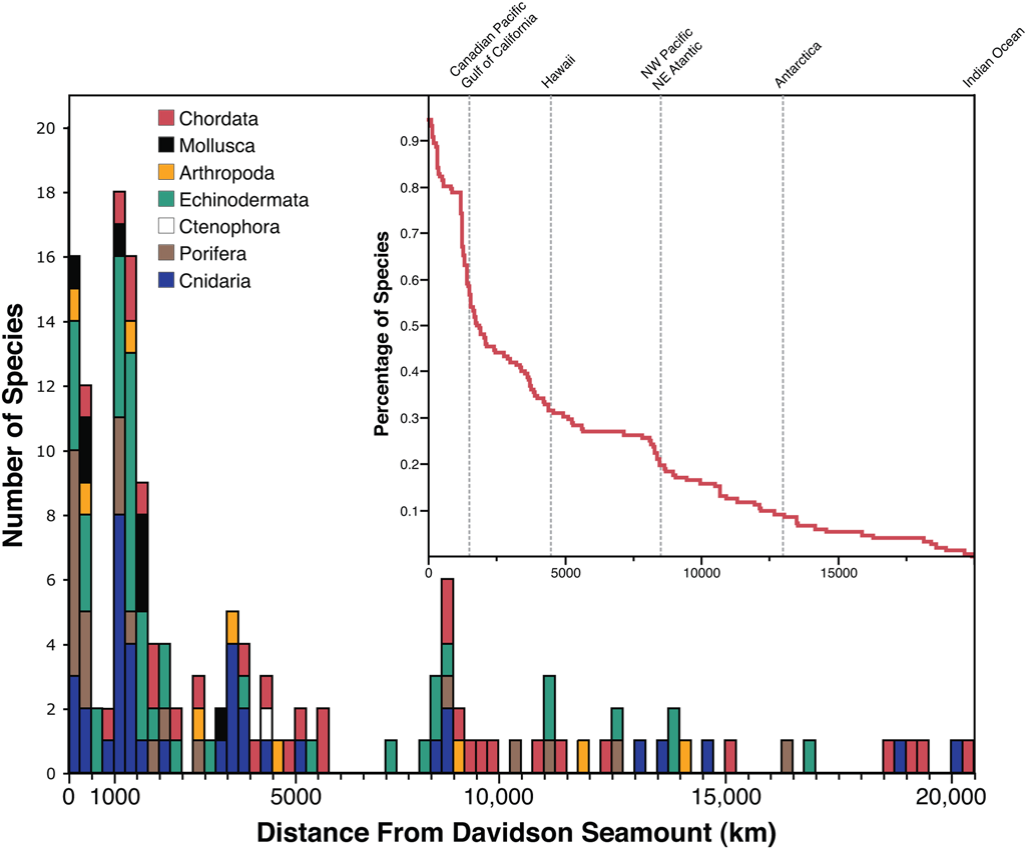
Frequency distribution of geographic ranges sizes as linear distance from Davidson Seamount. Colors denote varying contribution of different animal phyla. Subplot is the inverse cumulative frequency distribution of range sizes. Percentages denote species with ranges sizes greater than range (*x*).

Overall, our results indicate that species with large ranges (>1000 km) dominate the fauna of Davidson Seamount (Fig. 4). Seventy-nine percent of observed species have ranges that extend at least 1000 km from Davidson with 50% of the fauna greater than 1800 km. Several species have ranges that extend from the Gulf of California to the Northeast Pacific Ocean off Canada, the extent of the California current. A major break in the probability distribution (Fig. 4) occurs at this spatial scale (∼1500 km). It is important to note, however, that our dataset relies heavily on MBARI research efforts concentrated in this area. Another sudden shift occurs around 3500–4500 km, the distance from Davidson to Hawaii, and the furthest western extent of MBARI's sampling. The break at 8500 km represents records extending to the Northwest Pacific, possibly indicating a ‘Ring of Fire” Pacific distribution. A small minority of this group also includes species found in the Atlantic Ocean with greater geographic ranges, an artifact of the conservative linear approach we utilize (see methods). Amazingly ∼10% of the fauna at Davidson have ranges greater than 13000 km extending into either the Antarctic or Indian Oceans. Taxonomically, the largest faunal components of Davidson, the cnidarians (typically, deep-sea corals), poriferans, and echinoderms[21], account for the majority of the smaller ranges. Those species with ranges less than 500 km include the 12 unresolved species mentioned above. The remaining species are those with ranges spanning a minimum distance from Davidson Seamount to Monterey Canyon and often to nearby seamounts such as Rodriguez and Pioneer.

**Figure 5:**
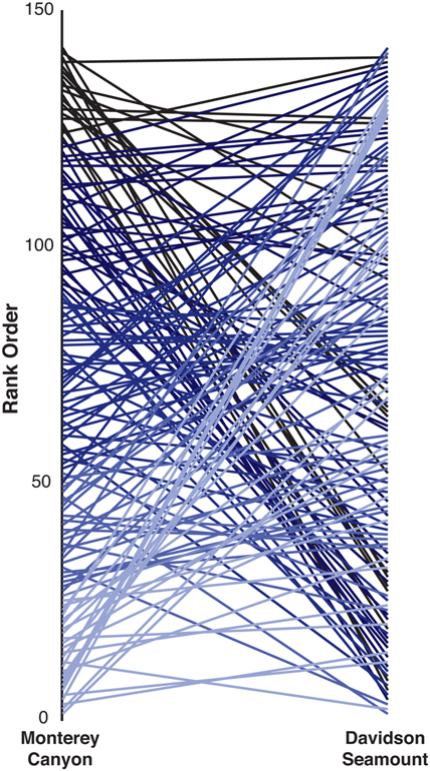
Rank orders based on frequency of observations in Monterey Canyon and Davidson Seamount. Lines connect the rank orders of a species at the two localities.

A course analysis of the rank order of species based on their frequency of observation in Monterey Canyon and Davidson Seamount indicates the communities, while sharing high overlap in species composition, are structurally quite different. Species that are relatively rare in Monterey Canyon are the most dominate at Davidson, and vice versa (Fig. 5).

## Discussion

In analyzing an entire megafaunal assemblage, we find little evidence to support the seamount endemicity hypothesis (SMEH)[14]. Our sampling curve shows Davidson Seamount is relatively well sampled, indicating that any undiscovered endemic species are likely to be rare. Most species are found on other seamounts and non-seamount habitats, and nearly all of the small percentage of potentially endemic species are rare and have unclear species assignments. It should be noted that a majority of the species here are not restricted to just Davidson or even to seamount habitats. The number of species potentially confined to seamounts in this study is 12%, those species potentially confined to Davidson is 7%, and those species observed less than ten times is 9%. If these percentages are assumed to represent the actual level of endemicity, they are still low compared to other unique deep-sea habitats or true island communities with rates often higher than 75%[18]. One caveat of the study, like many deep-sea studies, is that species identifications are based solely on morphological taxonomy and that species here could represent cryptic species. Further work using molecular methods will be needed to validate this work.

Seamount endemicity is posited to result from either geographic isolation, hydrodynamic features that trap larvae, or the presence of unique habitats rarely encountered only rarely in the background deep sea. The lack of endemicity on Davidson Seamount implies that either these mechanisms are insufficient to isolate seamount faunas or not applicable to all seamounts. In either case, SMEH is not a general rule broadly applicable to all seamounts. Wilson and Kaufman[15] noted previously that seamounts deeper and closer to the continental margin would possess a greater percentage of “widespread to cosmopolitan species”. Given Davidson's depth (∼1250–3600 m) and proximity to shore (120 km), our analyses appear to support this assertion. Although further work is required to determine whether endemism is greatest on more geographically isolated seamounts, O'Hara[19] and Samadi et al.[20] reported recently that endemism is not particularly high on seamounts separated by distances of 100 km to over 1000 km from the slope. The summit of Davidson is characterized by coral/sponge fields with a vast majority of the seamount comprised of hard substrate. This rugged hard substratum habitat is markedly different than the soft-sediment seabed that dominates most of the surrounding abyssal plain, continental rise, and slope. Even though habitats with hard substrata are rare and patchy in the region, except on the seamount, biogeographic isolation does not appear to be common. Previous work provides some evidence that Taylor columns form over Davidson, which also affects meander formation and persistence as the California Current and Undercurrent flow past the seamount. Again the lack of endemicity at Davidson Seamount implies that these hydrodynamic processes are insufficient to trap larvae [22].

The frequency distribution of range sizes for species found at Davidson Seamount is similar to the unimodal, right-skewed distribution observed for other faunas[6], [7], [23]. The shape of this distribution should be interpreted with caution since ranges are linear, representing distances from Davidson Seamount that may not be the center of the range, and are severely undersampled. New information concerning each of these factors can only increase the known biogeographic range size for individual species, thereby shifting the overall distribution of ranges toward more normal or left skewed. Therefore, the existing dataset represents a liberal estimate of the actual rate of endemicity, and new data (barring the discovery of presently unknown endemic species) will weaken support for the ‘seamount endemicity’ hypothesis. What the analysis suggests, despite the caveats discussed, is that the ranges of seamount organisms like other deep-sea taxa are large, often extending 1000's of kilometers. Many of the species here have ranges spanning the Pacific Ocean from the Northeast Pacific to Hawaii, the Bering Sea, the Sea of Japan, and in some cases to Antarctica. Some species may even be considered to have global distributions encompassing the Pacific, Atlantic, Indian, and Polar Oceans.

Although the Davidson Seamount megafaunal assemblage is compositionally similar to other deep-sea environments, we provide preliminary evidence that seamount communities are vastly different. Those species we observe rarely at Davidson Seamount are encountered frequently in nearby Monterey Canyon. Those species that dominate the Davidson megafauna assemblage are encountered rarely in the canyon. Indeed, the rank orders in Figure 5 display a remarkable reversal in the rank order of species between the two habitats. Our observations from Davidson show that summit assemblages contain dense aggregations of corals and sponges. These species are encountered at similar depths along the rocky walls of Monterey Canyon, but at far lower densities or dominance than occurs at Davidson.

*Our observations support the notion that although endemicity may not be a key feature of seamount communities, they are structurally different than most other deep-sea communities*.

Reasons for differences in observed community structure may range from contrasts in disturbance regimes, type and quantity of substrate, flow regimes that favor particular trophic guilds, or organic input[15], [17], [18], [24]. Seamount environments may represent optimal habitats for particular faunal groups resulting in thriving and dense populations encountered only rarely in other habitats. In this scenario, seamount assemblages are likely to be sources of larvae that maintain populations of certain species in sub-optimal, non-seamount sinks[25]. A similar source-sink system has been proposed for bathyal and abyssal systems, driven by the exponential decrease in carbon flux that results in markedly contrasting food availability between the two systems[13]. We caution that both the ‘seamount structure’ and the ‘seamount source-sink’ hypotheses remain speculative and require further testing. Future studies using careful experiments or analyses to control for depth, substrate type, and sampling area will be required to quantify structural differences between seamount and non-seamount habitats and clarify processes regulating these patterns. As with the source-sink hypothesis for abyssal biodiversity[13], investigations examining genetic population structure are likely to provide the strongest tests.

Seamount conservation has recently received much attention[26], [27]. The perceived endemicity, presence of long-lived, slow-growth corals and sponges, and dense aggregations of commercially important fishes, may make seamounts particularly vulnerable to various stressors[28]–[31]. Because the SMEH differs from other hypotheses explaining faunal organization on seamounts (e.g., the source-sink and oasis[20] hypotheses), management and policy implications of these hypotheses should be considered carefully[18]. These hypotheses contrast sharply – the SMEH implies that ecological and evolutionary processes on seamounts are largely disjunct from those operating in adjacent habitats. These others postulate that seamounts are sources of larvae for surrounding areas and are therefore integrated broadly in the biological landscape. Each of these scenarios, high endemicity, high biodiversity, or local source populations for larvae, justify the protection and conservation of seamount resources.

Overall, we find little support for the SMEH and instead document a seamount assemblage dominated by cosmopolitan species. As our study and many others have focused exclusively on megafauna, future work is required to examine the extent that macro- and meiofauna follow SMEH. Our preliminary results do suggest that structure of seamounts assemblages may differ from other deep-sea benthic habitats and may prove to be source populations for many deep-sea species. Though speculative at this time, we are excited by the potential of these new hypotheses to guide future research and refine our understanding of deep-sea processes. Although each of these hypotheses has important policy and conservation implications, much research is still needed and we advise caution in incorporating them into seamount conservation strategies.

## Methods

In this report, we focus on megafauna animals, those organisms readily identifiable in video or caught in trawls. We constructed a faunal inventory for Davidson Seamount, the first bathymetric feature to be characterized as a ‘seamount’, residing 120 km off the central California coast in approximately 3600 m of water (Fig 1). Davidson Seamount rises approximately 2400 m off the surrounding abyssal plain. Similar to other local seamounts, Davidson has volcanic origins (9–16 mya). The seamount comprises a series of southwest to northeast trending ridges interspersed with cones and sediment troughs. At 42 km in length, 13 km in width, and with its substantial elevation, Davidson Seamount ranks as one of the largest seamounts in U.S. waters.

Between 2000–2007, five oceanographic expeditions including remotely operated vehicle (ROV) dives visited Davidson Seamount. All expeditions were conducted with the research vessel *Western Flyer* and the ROV *Tiburon* by the Monterey Bay Aquarium Research Institute (MBARI), twice in collaboration with the Monterey Bay National Marine Sanctuary. In total, 28 ROV dives yielded over 60000 faunal observations on over 200 hours of video. All ROV dive video has been reviewed in detail using MBARI's Video Annotation and Reference System[32]. This system represents a knowledge database of all biological, geological, technological objects observed on any ROV dive made by MBARI over the last 26 years. The database can be queried with different search terms (e.g., various taxonomic levels) and constrained by a variety of parameters (e.g., location and depth) and thus provides information about the biogeographic ranges within the extent of MBARI's exploration. Additional information about biogeographic ranges was culled from the literature, Seamounts Online, and FishBase.

All megafauna were identified to the species level or equivalent (e.g., *Calyptrophora* sp. 1) by trained video annotators using *in situ* video frame grabs and/or voucher specimens that were identified by taxonomists. In total, 225 voucher specimens were sent out for identification by taxonomic experts. Although every effort was made to assign organisms that were morphologically distinct (i.e., morphospecies) an appropriate Latin bionomial, 38% of the species possess identifying tags above the species level (e.g., *Calyptrophora* sp. 1). The following taxonomic experts were consulted with: R. Lee for Actinopterygii, G. Cailliet for Actinopterygii, D. Pawson for Holothuroidea, G. Rouse for Polychaeta, S. Cairns for Octocorallia, H. Reiswig for Hexactinellida, W. Lee for Demospongiae, G. Williams for Octocorallia, C. Mah for Asteroidea, C. Messing for Crinoidea, and R. Mooi for Echinoidea. Voucher specimens were not collected for organisms that could be identified easily from video and are known from the California shelf and Monterey Canyon. In some instances, voucher specimens were not obtainable and video frame grabs, digital still images, and/or video segments of the organisms in question were reviewed by taxonomists with expertise in that particular taxonomic group. Species identifications primarily relied on recently collected high-definition video collected since 2006. Recently, the usage of video and still images in biogeographic, ecological, and taxonomic studies of deep-sea species has become common and vital tool for describing both species and faunas in habitats logistically difficult to sample like the deep sea[21], [33]–[36].

We report endemicity (i.e., a species restricted to Davidson Seamount) based on information gathered from the above sources. Species were assigned an endemicity certainty code (ECC) based on the evidence of their occurrence off Davidson. Species assigned an ECC of 1 were considered to have enough supporting data to *indicate they are not endemic* (i.e., ranges are confirmed by taxonomic specialists). Species of an ECC of 2 have enough support to *suggest reasonably that they are not endemic* (i.e., ranges are based on morphologically similar species identified in video). Species of an ECC of 0 are known only from Davidson Seamount, and *are potential endemics*. Estimates of shared species between central California seamounts was taken from Lundsten[37].

We ranked species abundance overall and among habitat types according to their rarity, determined from the number of observations of a species on Davidson Seamount and in nearby Monterey Canyon. A comparison of rarity among habitats was made by plotting the ranks of species in the two localities. Geographic range was calculated as the maximum linear distance (in km) from Davidson Seamount for the most conservative identifications. Coordinates were transformed into linear distance by assuming the earth is a perfect sphere with a radius of 6378 km. For species occurring on Davidson Seamount in the Pacific Ocean and also in the Atlantic Ocean, we assume the distance between sampling sites is a straight line (i.e., across North America). We do this as information between sampling localities may be lacking that would indicate a specific route of range expansion. Note that this biases species toward smaller range sizes. Species accumulation curves were calculated in EstimateS with all other analyses conducted in JMP Statistical Software v. 5.
